# Dye-Decolorizing Peroxidase of *Streptomyces coelicolor* (*Sc*DyPB) Exists as a Dynamic Mixture of Kinetically Different Oligomers

**DOI:** 10.1021/acsomega.3c07963

**Published:** 2024-01-08

**Authors:** Hegne Pupart, Darja Vastšjonok, Tiit Lukk, Priit Väljamäe

**Affiliations:** † Department of Chemistry and Biotechnology, Tallinn University of Technology, 15 Akadeemia tee, Tallinn 12618, Estonia; ‡ Institute of Molecular and Cell Biology, University of Tartu, Riia 23b-202, Tartu 51010, Estonia

## Abstract

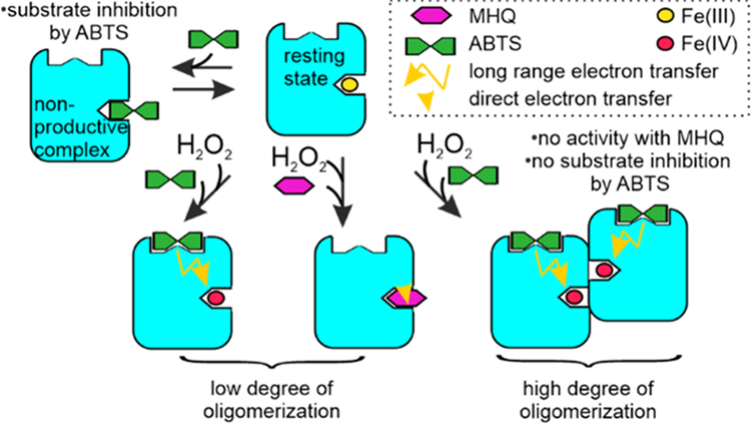

Dye-decolorizing peroxidases (DyPs) are heme-dependent enzymes that catalyze the oxidation of various substrates including environmental pollutants such as azo dyes and also lignin. DyPs often display complex non-Michaelis–Menten kinetics with substrate inhibition or positive cooperativity. Here, we performed in-depth kinetic characterization of the DyP of the bacterium *Streptomyces coelicolor* (*Sc*DyPB). The activity of *Sc*DyPB was found to be dependent on its concentration in the working stock used to initiate the reactions as well as on the pH of the working stock. Furthermore, the above-listed conditions had different effects on the oxidation of 2,2′-azino-di(3-ethyl-benzothiazoline-6-sulfonic acid) (ABTS) and methylhydroquinone, suggesting that different mechanisms are used in the oxidation of these substrates. The kinetics of the oxidation of ABTS were best described by the model whereby *Sc*DyPB exists as a mixture of two kinetically different enzyme forms. Both forms obey the ping-pong kinetic mechanism, but one form is substrate-inhibited by the ABTS, whereas the other is not. Gel filtration chromatography and dynamic light scattering analyses revealed that *Sc*DyPB exists as a complex mixture of molecules with different sizes. We propose that *Sc*DyPB populations with low and high degrees of oligomerization have different kinetic properties. Such enzyme oligomerization-dependent modulation of the kinetic properties adds further dimension to the complexity of the kinetics of DyPs but also suggests novel possibilities for the regulation of their catalytic activity.

## Introduction

Dye-decolorizing peroxidases (EC 1.11.1.19, DyPs) are enzymes that catalyze the oxidation of various substrates using H_2_O_2_ as an electron acceptor. Although the physiological substrate has not been identified yet, these peroxidases are known to oxidize anthraquinone and azo dyes,^1^ β-carotene,^2^ and different phenolic compounds^3^ including lignin. There has been a growing interest in DyPs due to their biotechnological potential in bioremediation of industrial dyes and valorization of lignin.^4^

To date, over 50 different DyPs have been characterized.^5,6^ These heme-dependent peroxidases are present in both fungal and bacterial species, and the family of DyPs is divided into four subfamilies (type A, B, and C/D) according to their primary sequences (RedOxiBase) with a sequence identity less than 15% between the subfamilies.^7−10^ All DyPs adopt a similar dimeric ferredoxin-like fold consisting of β-sheets and peripheral α-helices, distinct from well-known peroxidases, such as horseradish peroxidase.^11^ DyPs belong to the CDE superfamily, a part of the “dimeric α + β barrel superfamily”.^12−14^ Heme is held in the heme cavity, positioned in the C-terminal domain of the monomer, where it is ligated by a proximal conserved histidine residue.^9,15^

Being able to degrade bulky textile dyes and even polymeric lignin, DyPs differ from classic peroxidases.^4,8,9,16^ However, like other heme peroxidases, DyPs obey the ping-pong kinetic mechanism, by cycling between the ferric resting state and its high-valent intermediates compound I (Cpd I) and compound II (Cpd II).^8^ Smaller reducing substrates as well as the H_2_O_2_ cosubstrate can access the heme through channels that connect heme with the protein exterior.^17,18^ To catalyze the oxidation of substrates with a bulkier size, DyPs are known to apply long-range electron transfer pathways. In this case, the electrons are first transferred from the reducing substrate to the solvent-accessible amino acid residues at the surface (usually Trp or Tyr residues) from where they are further transferred to the high-valent intermediate of heme.^19,20^

Like for other heme peroxidases, the general ping-pong kinetic mechanism of DyPs is well studied and generally accepted. At the same time, the molecular mechanisms behind the non-Michaelis–Menten kinetics that are often observed with DyPs and characterized by the presence of substrate inhibition^18,21−26^ and cooperative effects^15,22,27,28^ remain largely unknown. The oligomeric state of DyPs varies from monomers to hexamers. Most of the fungal DyPs are monomeric;^1,22,29−33^ however, the existence of dimers has also been described.^2,34^ It has been suggested that loop insertions and the increasing complexity of fungal DyPs favor the monomeric form by hindering the oligomerization.^11,35^ In contrast to their fungal counterparts, the bacterial DyPs exist mostly as oligomers, including dimers,^11,28,35−41^ tetramers,^26,42,43^ pentamers,^44^ and hexamers.^11,45,46^ Nevertheless, some bacterial DyPs have been shown to exist as monomers^3,26,36,47^ or as a mixture of enzyme populations with different degrees of oligomerization such as monomer and dimer^48−50^ and monomer and oligomer (*n* = 4–6).^51−53^ The DyP from *Thermomonospora curvata* was shown to exist as a mixture of dimer, tetramer, and octamer.^27^ Furthermore, many bacterial DyPs are often loaded as cargo proteins into the encapsulin nanoparticles,^45,46,53−55^ where they exist at an even higher degree of oligomerization.^54^ Although it has been shown that the DyP–encapsulin complex has higher lignin degrading activity compared to nonencapsulated DyP^45^ and that monomeric forms may be deficient in the binding of heme,^50,52,53^ little is known about the relations between the degree of oligomerization of DyPs and their catalytic activity.

Here, we performed an in-depth kinetic characterization of the subfamily B DyP of the bacterium *Streptomyces coelicolor* (*Sc*DyPB) along with analyses of its size distribution. Our results suggest that *Sc*DyPB exists as a mixture of enzyme forms with different degrees of oligomerization, which differed in their catalytic properties.

## Materials and Methods

ABTS (lot no. SLBT0759) and methylhydroquinone MHQ (lot no. BCBH9920 V) were purchased from Sigma-Aldrich. BSA (lot no. K00113-2235, Fraction V) was obtained from GE Healthcare. The concentration of the H_2_O_2_ stock solution (Honeywell, lot # SZBG2070) was determined spectrophotometrically at 240 nm using an extinction coefficient of 39.4 M^–1^ cm^–1^.^56^ Dilutions of a H_2_O_2_ stock solution were prepared in water before use. Milli-Q (mQ) ultrapure (type 1) water was used in all experiments.

### Recombinant *Sc*DyPB

Dye-decolorizing peroxidase from *S. coelicolor* A3(2) (*Sc*DyPB, UniProtKB Q9FBY9) was overexpressed in *Escherichia coli* BL21 (DE3), purified, and reconstituted with hemin as described in Pupart et al.^23^ The concentration of *Sc*DyPB was determined by the absorbance of the heme at 406 nm using an extinction coefficient of 100,000 M^–1^ cm^–1^ or by the absorbance at 280 nm using an extinction coefficient of 18,450 M^–1^ cm^–1^. The concentration of the enzyme storage stock was 18.1 ± 0.6 μM (based on the absorbance at 406 nm) and 39.0 ± 1.6 μM (based on the absorbance at 280 nm). In all experiments, the enzyme was dosed based on its concentration measured by the absorbance of heme. *Sc*DyPB was stored at −80 °C as frozen aliquots in 20 mM Tris-HCl (pH 7.5) containing 0.1 M NaCl. *Sc*DyPB working stocks were made by the dilution of the storage stock to the appropriate buffer containing 0.1 g L^–1^ BSA and 0.1 M NaCl.

### Measuring the Activity of *Sc*DyPB

ABTS and MHQ were used for the kinetic characterization. If not stated otherwise, the activity measurements were performed in 50 mM sodium acetate (pH 4.0) in a spectrophotometer (Shimadzu UV-1900i UV–vis) cuvette at 25 °C in a total volume of 1.0 mL. The oxidation of ABTS was measured by the increase in the absorbance at 420 nm (ε_420_ = 36,000 M^–1^ cm^–1^) and that of MHQ by the increase in the absorbance at 251 nm (ε_251_ = 21,450 M^–1^ cm^–1^).^57^ Reactions were started by the addition of the enzyme from its working stock to a cuvette containing a mixture of the reducing substrate and H_2_O_2_. The nonenzymatic oxidation of substrates was measured in the experiments without the enzyme, and all activity measurements were corrected for this background. If not stated otherwise, the oxidation of ABTS was measured using 1 mM ABTS and 100 μM H_2_O_2_, and the oxidation of MHQ was measured using 1 mM MHQ and 1 mM H_2_O_2_. The oxidation of textile dyes reactive blue 4 (RB4) and 19 (RB19) was tested using 0.15 μM *Sc*DyPB, 0.1 mM, dye, and 1.0 mM H_2_O_2_ in 50 mM NaAc (pH 4.0) at 25 °C. Oxidation of the RB4 was measured by the decrease of the absorbance at 610 nm using an ε_610_ of 4200 M^–1^ cm^–1^ and by that of RB19 at 595 nm using an ε_595_ of 10,000 M^–1^ cm^–1^. The data were analyzed by using STATISTICA 8.0 and GraphPad Prism 5 software.

### Inactivation of *Sc*DyPB by H_2_O_2_

1.5 μM *Sc*DyPB in 50 mM NaAc (pH 4.0) or 20 mM Tris-HCl (pH 7.5) (both supplemented with 0.1 g L^–1^ BSA) was preincubated with H_2_O_2_ (0–5 mM) at 25 °C. At selected times, a 10 μL aliquot was withdrawn and added to the cuvette containing 990 μL of the mixture of 1 mM ABTS and 0.1 mM H_2_O_2_ and the activity was measured by the increase in the absorbance at 420 nm.

### Gel Filtration Chromatography

A Superdex 75 Increase 10/300 GL column (GE Healthcare) was equilibrated with 20 mM Tris-HCl and 0.1 M NaCl, pH 7.5. 100 μL of 18.1 μM *Sc*DyPB was injected, and the column was eluted with equilibration buffer at a flow rate of 0.5 mL min^–1^. Elution of the proteins was monitored by the absorbance at 280 nm. The high-molecular weight gel filtration calibration kit (Cytiva) contained ovalbumin (43 kDa), conalbumin (75 kDa), aldolase (158 kDa), and ferritin (440 kDa). The standard proteins were dissolved in Tris-HCl buffer (20 mM, pH 7.5, supplemented with 0.1 M NaCl) and used for calibration.

### Dynamic Light Scattering

Dynamic light scattering (DLS) analyses were performed using a Zetasizer Nano S particle size analyzer (Malvern Panalytical) with a constant 173 C scattering angle at 25 °C and the laser wavelength of 633 nm. Before DLS analysis, the buffers (20 mM Tris-HCl, pH 7.5, 0.1 M NaCl, or 50 mM NaAc mM, pH 4.0) were filtered through a sterile syringe filter with 0.22 μm pore size. The sample volume used for analysis was 80 μL, and the concentration of *Sc*DyPB was 1.5 μM. The scattering intensity data were processed using instrumental software to obtain the hydrodynamic diameter (*D*_h_) and the size distribution of scatters in each sample.

### Spectra of *Sc*DyPB

The UV–vis absorption spectra of *Sc*DyPB were recorded at 250–700 nm using a Shimadzu UV-1900i UV–vis spectrophotometer at 25 °C. After recording the spectrum of 3.6 μM *Sc*DyPB in 4 mM Tris-HCl (pH 7.5, supplemented with 20 mM NaCl) in 0.5 mL total volume, the pH was brought to 4.0 by the addition of 26 μL of 1.0 M NaAc (pH 4.0) and spectra were recorded again. Finally, 150 μL of 1.0 M Tris-HCl (pH 7.5) was added, and spectra were recorded.

## Results

### Measuring the Activity of *Sc*DyPB

**Scheme 1:**
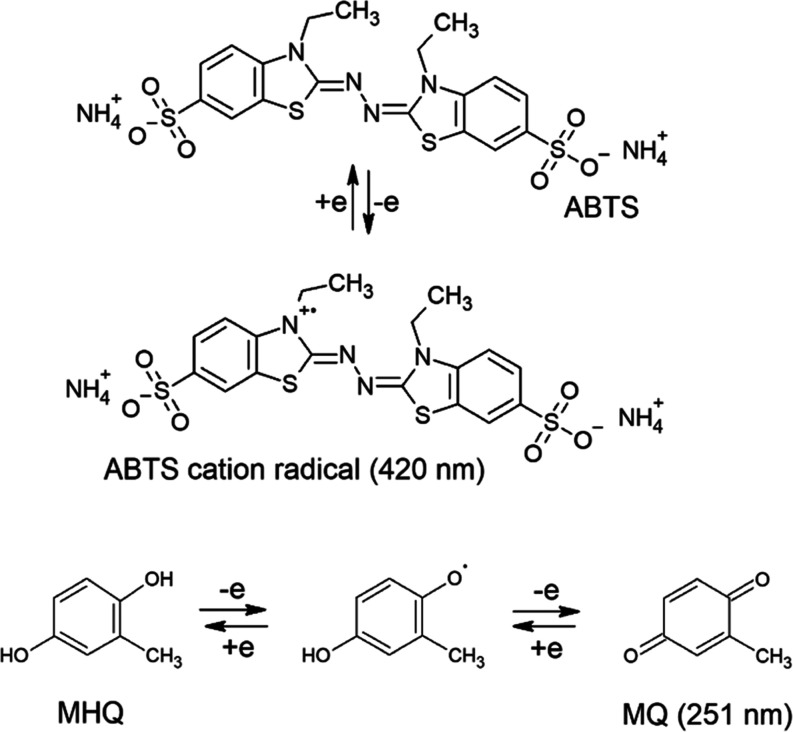
Oxidation of 2,2′-Azino-di-(3-ethyl-benzothiazoline-6-sulfonic acid) (ABTS) and methylhydroquinone (MHQ) One-electron oxidation of ABTS to the ABTS cation radical and two-electron oxidation of MHQ to methylquinone (MQ) (absorption maxima at 420 and 251 nm, respectively).

Kinetic characterization of *Sc*DyPB was performed using two reducing substrates: a conventional peroxidase substrate, 2,2′-azino-di(3-ethyl- benzothiazoline-6-sulfonic acid) (ABTS), and a phenolic substrate, methylhydroquinone (MHQ). ABTS is a one electron-donating substrate, and its oxidation can be followed by the absorbance of the ABTS cation radical product (ABTS^+•^) at 420 nm. MHQ is a two electron-donating substrate, and its oxidation can be followed by the absorbance of the methylquinone (MQ) product at 251 nm (Scheme 1).

**Figure 1:**
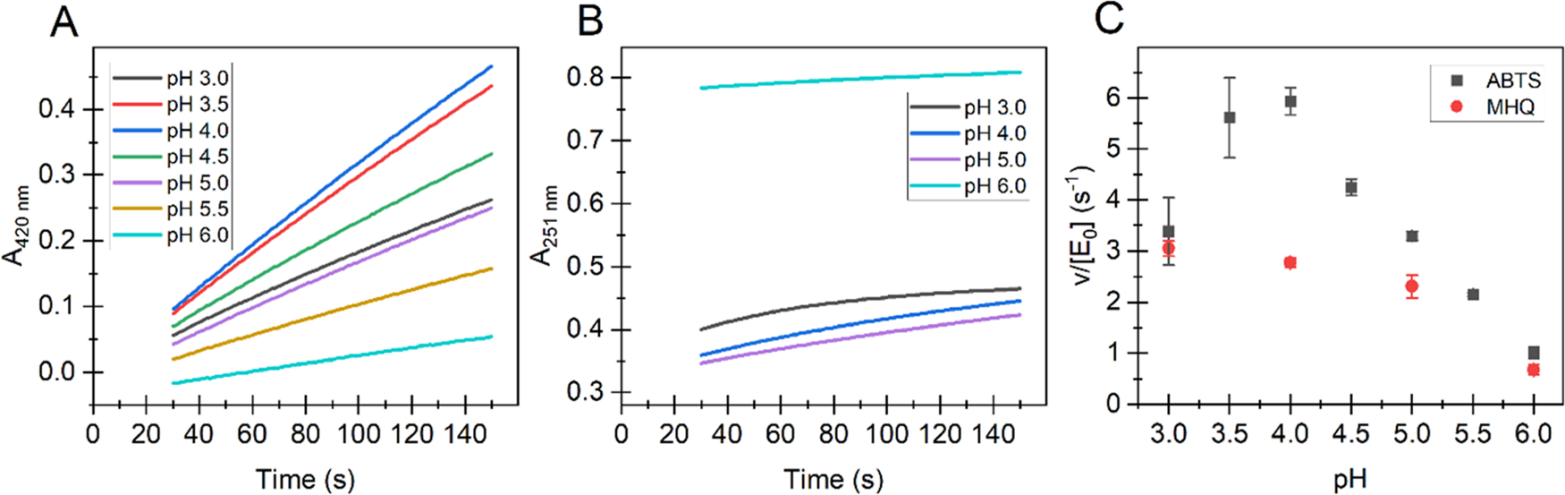
Dependency of initial rates of reducing substrate oxidation on pH by *Sc*DyPB. The time curves of the oxidation of (A) ABTS and (B) MHQ. The pH values are given in the plot. (C) Dependency of the initial rates (measured between 30 and 60 s) of the oxidation of ABTS and MHQ on pH. All reactions were performed at 25 °C. Buffers were 50 mM sodium citrate (for pH 3.0 and 3.5), 50 mM sodium acetate (for pH 4.0–5.0), and 50 mM Bis–Tris–HCl (for pH 5.5 and 6.0). Reactions were initiated by the addition of the *Sc*DyPB from its working stock (1.5 μM *Sc*DyPB in 20 mM Tris-HCl pH 7.5 supplemented with 0.1 g L^–1^ BSA and 0.1 M NaCl) to the cuvette containing the mixture of the substrate and H_2_O_2._ The final concentration of the *Sc*DyPB in the cuvette was 15 nM. The oxidation of ABTS was measured using 1 mM ABTS and 100 μM H_2_O_2_. The oxidation of MHQ was measured using 1 mM MHQ (note that MHQ preparation had a high background absorbance at pH 6.0) and 1 mM H_2_O_2_. Data are presented as average values (*n* = 3, independent experiments), and error bars show SD. For clarity, the error bars are not shown for the traces in panels (A) and (B).

All peroxidase reactions were carried out in a spectrophotometer cuvette at 25 °C. Reactions were started by the addition of the enzyme from its working stock to the cuvette containing the mixture of the reducing substrate and H_2_O_2_, and all rates correspond to the initial rates (measured between 30 and 60 s, Figure 1A,B). The pH optima of the oxidation of both substrates were around 4 (Figure 1C), and all further activity measurements were made in 50 mM sodium acetate (NaAc), pH 4.0.

### Dependency of the Activity on the Concentration of *Sc*DyPB

**Figure 2:**
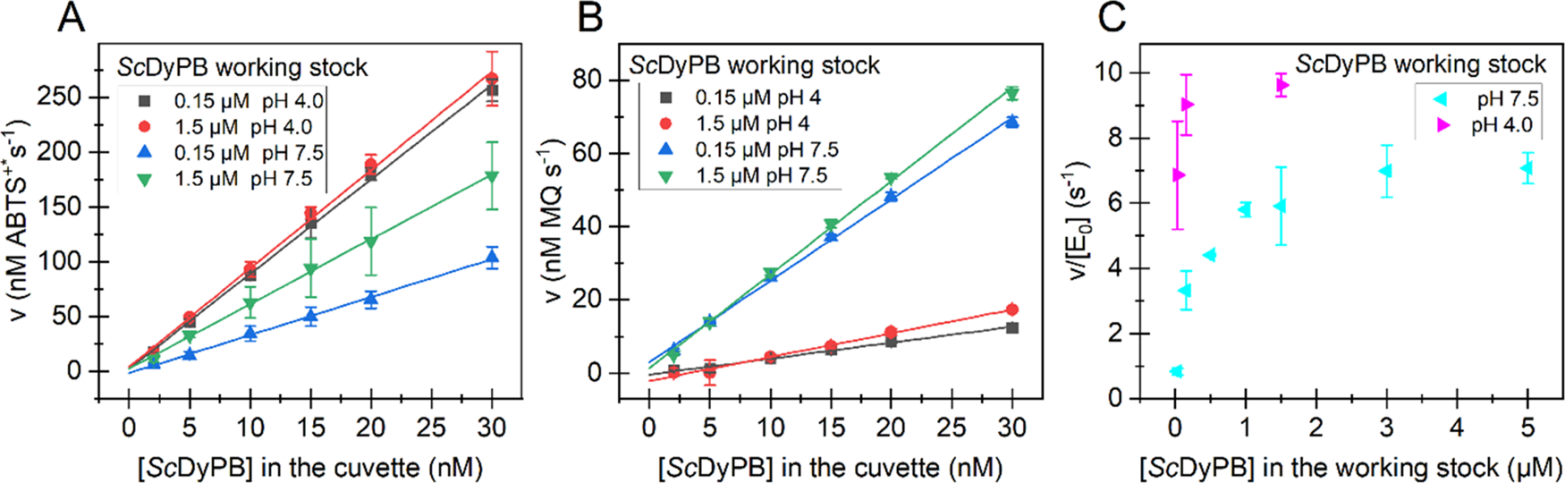
Dependency of the activity on the concentration of *Sc*DyPB in the cuvette and in its working stock. All reactions were performed in NaAc buffer (50 mM, pH 4.0) at 25 °C. The oxidation of ABTS was measured using 1 mM ABTS and 100 μM H_2_O_2_ and the oxidation of MHQ with 1 mM MHQ and 1 mM H_2_O_2_. The rates of oxidation of (A) ABTS and (B) MHQ at different concentrations of *Sc*DyPB in the cuvette. The concentration of *Sc*DyPB in its working stock and the pH of the working stock are shown in the figure. Solid lines show the linear regression of the data. (C) Dependency of the ABTS oxidizing activity of *Sc*DyPB on its concentration in the working stock. The pH of the *Sc*DyPB working stock is shown in the figure. The concentration of the *Sc*DyPB in the cuvette was 15 nM. The working stocks of *Sc*DyPB, with concentrations of 30 nM–5 μM, were prepared in 20 mM Tris pH 7.5, 0.1 M NaCl, or 50 mM NaAc pH 4.0 buffers (both supplemented with 0.1 g L^–1^ BSA). Data are presented as average values (*n* = 3, independent experiments), and error bars show SD.

As expected for the enzyme-catalyzed reactions, the rate of the oxidation of both ABTS and MHQ scaled linearly with the concentration of the *Sc*DyPB in the cuvette (Figure 2A,B). However, we found that the activity of *Sc*DyPB was dependent on its concentration in the working stock used for the initiation of the enzyme reactions in the cuvette as well as on the pH of the working stock. Furthermore, the presence and the direction of these effects were dependent on the substrate used for the activity measurements. With ABTS as the substrate, the activity of the enzyme from the working stock made in NaAc pH 4.0 was about 1.5–2.5-fold (depending on the concentration of *Sc*DyPB in the working stock) higher than that from the working stock made in Tris pH 7.5 (Figure 2A). The opposite was true for the activity measured with MHQ where the reactions that started from the working stock made in NaAc pH 4.0 had about sixfold lower activity compared to those made in Tris pH 7.5 (Figure 2B).

With ABTS as the substrate, we made a series of activity measurements using different *Sc*DyPB concentrations in the working stock. The activity seemed to reach a plateau value with an increasing concentration of *Sc*DyPB in the working stock (Figure 2C). An apparent half-saturating concentration of *Sc*DyPB in the working stock made in 20 mM Tris pH 7.5 was about 0.38 μM, whereas the corresponding figure for the working stock made in 50 mM NaAc pH 4.0 was below 0.03 μM (Figure 2C). We note that in all cases, the concentration of *Sc*DyPB in the cuvette was 15 nM. The simplest explanation for this phenomenon would be a nonspecific binding of *Sc*DyPB to the laboratory plastics, like microcentrifuge tubes used for preparing enzyme working stocks. The results of the preliminary experiments, indeed, suggested that such nonspecific binding exists but not in the presence of BSA that was present (at 0.1 g L^–1^) in the working stocks of *Sc*DyPB used in all experiments shown in this work. Furthermore, one may expect that the nonspecific binding to laboratory plastics would have similar effects on the activities measured using different substrates such as ABTS and MHQ, which was clearly not the case in this study (Figure 2A,B).

The higher activity measured in the reactions that started from the working stocks with higher enzyme concentrations can be explained by the enzyme being active as an oligomer. In the case of reversible binding, the relative concentration of the oligomeric form is expected to increase with an increasing total concentration of the enzyme. However, the relaxation to a possible binding equilibrium between different oligomeric states must be slow enough not to be achieved during the activity measurements in the cuvette. The results of several control experiments made using different preincubation times of *Sc*DyPB working stocks suggested that the establishment of the possible new equilibrium upon the dilution of *Sc*DyPB working stocks was relatively slow (compared to the time frame of the activity measurement). Furthermore, higher activity was observed when ABTS was present in preincubation of the *Sc*DyPB working stock (Figure S1). These results suggest that equilibrium between different possible oligomeric forms of *Sc*DyPB that has been established in its working stock is at least partly retained during the activity measurement in the cuvette. Since higher total enzyme concentration is expected to favor the association, the higher activity observed in the case of higher concentration of *Sc*DyPB in its working stock (Figure 2C) suggests that higher oligomeric forms have higher ABTS oxidizing activity. If that is the case, the lower pH of the working stock seems to favor the higher oligomeric forms of *Sc*DyPB (Figure 2A,C). Although there was little dependency between the MHQ oxidizing activity and the concentration of *Sc*DyPB in its working stock, the lower activity observed with working stocks made at pH 4.0 (compared to those made in pH 7.5) (Figure 2B) suggests that contrary to the ABTS, the higher oligomeric forms have lower MHQ oxidizing activity. We also tested the oxidation of two textile dyes, RB4 and RB19, by *Sc*DyPB (using 0.1 mM dye and 1.0 mM H_2_O_2_), but the activity with these substrates was low with apparent rates of 0.66 ± 0.04 and 0.58 ± 0.02 s^–1^ for RB4 and RB 19, respectively.

### Effects of Additives on the Activity of *Sc*DyPB

**Figure 3:**
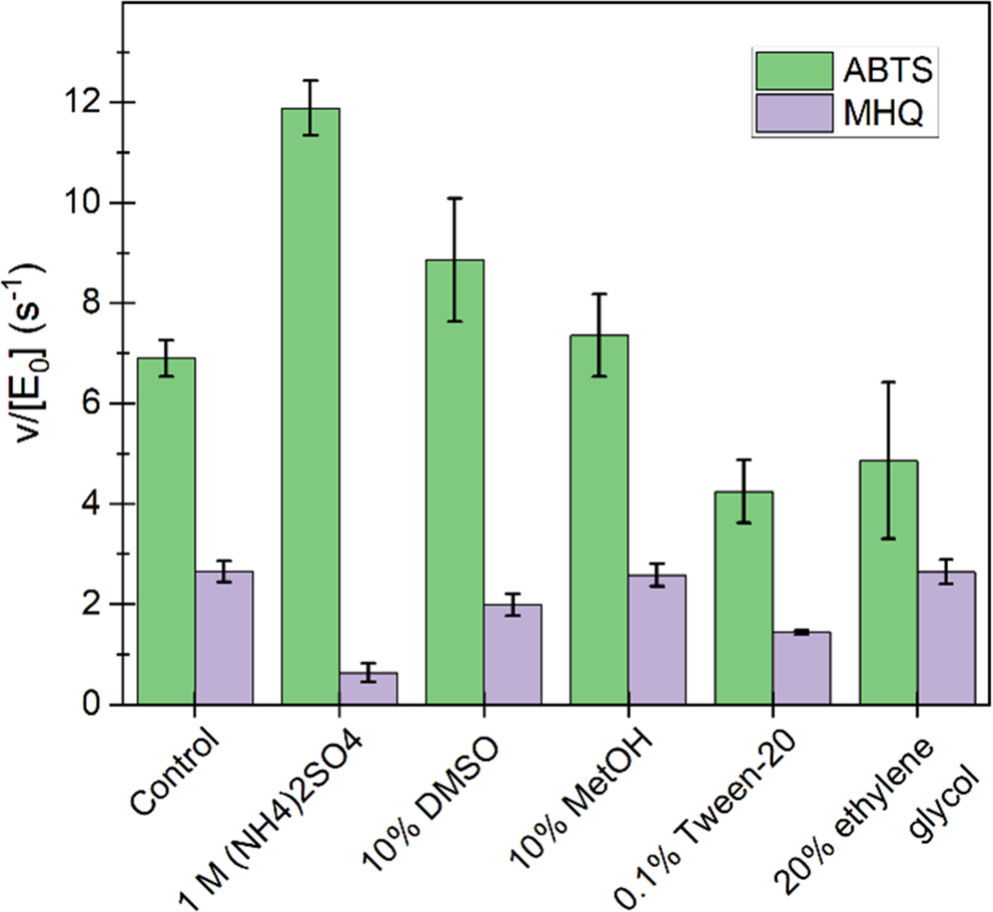
Effects of different additives in the working stock of *Sc*DyPB on the ABTS and MHQ oxidizing activity. Prior to the activity measurements, the *Sc*DyPB working stock (1.5 μM *Sc*DyPB in 20 mM Tris-HCl pH 7.5 supplemented with 0.1 g L^–1^ BSA and 0.1 M NaCl) was incubated for 30 min at 25 °C in the presence of different compounds as indicated in the figure. The activity was measured in 50 mM NaAc at pH 4.0 using 15 nM *Sc*DyPB (100-fold dilution of the working stock to the cuvette). The activity was measured using 1 mM ABTS and 100 μM H_2_O_2_ or 1 mM MHQ and 1 mM H_2_O_2_. Data are presented as average values (*n* = 3, independent experiments), and error bars show SD.

As shown above, the pH of the *Sc*DyPB working stock had differential effects depending on which substrate, ABTS or MHQ, was used for the activity measurement (Figure 2A,B). Here, we tested the effects of different additives (ammonium sulfate, DMSO, methanol, Tween-20, and ethylene glycol) in the *Sc*DyPB working stock (1.5 μM *Sc*DyPB in 20 mM Tris-HCl pH 7.5 supplemented with 0.1 g L^–1^ BSA and 0.1 M NaCl) to the ABTS and MHQ oxidizing activity. Among the compounds tested, only the presence of ammonium sulfate in the working stock of *Sc*DyPB had a significant effect on the activity (Figure 3). The presence of 1.0 M ammonium sulfate in the working stock resulted in about a twofold increase in ABTS oxidizing activity, whereas there was about a fourfold decrease in the MHQ oxidizing activity (Figure 3). Control experiments made with 0.01 M ammonium sulfate in the cuvette showed no effect on the activity (data not shown), confirming that the observed effects were caused by the presence of ammonium sulfate in the working stock of *Sc*DyPB. The opposite effects of ammonium sulfate on the activity with ABTS and MHQ corroborate with the effects of the pH of the working stock (Figure 2A,B) and suggest that different oligomeric forms of *Sc*DyPB may be responsible for the oxidation of ABTS and MHQ.

### Kinetics of the Oxidation of ABTS

**Figure 4:**
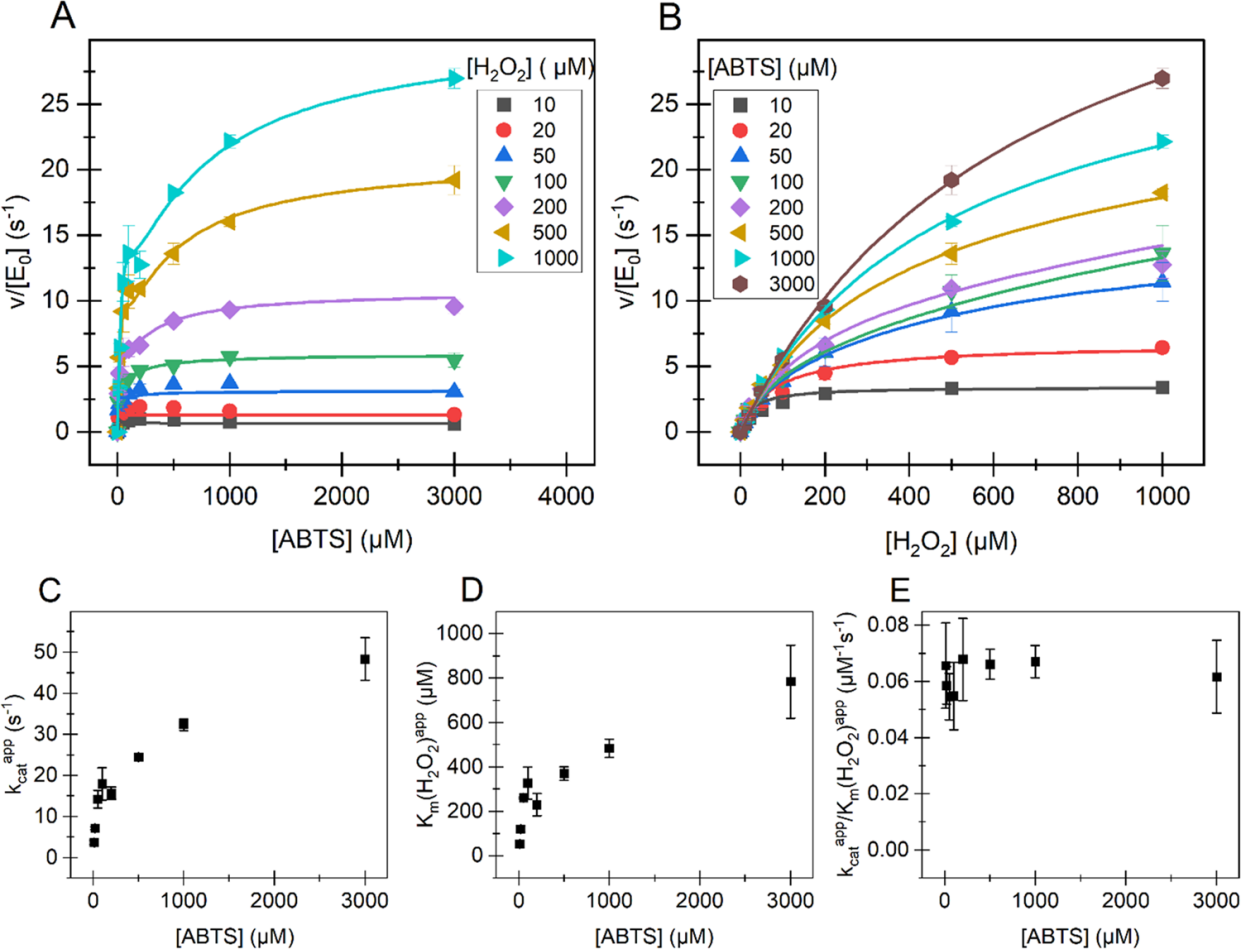
Kinetics of the oxidation of ABTS by *Sc*DyPB. Dependency of initial rates of the oxidation of ABTS on the concentration of (A) ABTS and (B) H_2_O_2_ and the dependency of apparent parameters (C) *k*_cat_^app^, (D) *K*_M__(H_2_O_2_)_^app^, and (E) *k*_cat_^app^/*K*_M__(H_2_O_2_)_^app^ on the concentration of ABTS. Reactions were made in 50 mM NaAc (pH 4.0) at 25 °C. The concentration of *Sc*DyPB was 15 nM, and the reactions were initiated by the addition of *Sc*DyPB from the working stock with 1.5 μM *Sc*DyPB in 20 mM Tris pH 7.5 (supplemented with 0.1 g L^–1^ BSA and 0.1 M NaCl). Solid lines in (A) and (B) show the global nonlinear regression of the data according to eq 2. The concentration of the substrate that has been kept constant within the series is indicated in the plot. The values of apparent kinetic parameters for H_2_O_2_ shown in panels (C–E) were derived by nonlinear regression analysis of the data in panel (B) according to the Michaelis–Menten equation (eq S1, for the fit, see Figure S2B). Data are presented as average values (*n* = 3, independent experiments), and error bars show SD.

All experiments described above were performed using ABTS and H_2_O_2_ concentrations of 1.0 and 0.1 mM, respectively. Here, we performed experiments with varied concentrations of ABTS (0.01–3.0 mM) and H_2_O_2_ (0.01–1.0 mM). The concentration of *Sc*DyPB in the cuvette was 15 nM, and the reactions were started by the addition of *Sc*DyPB from the working stock with 1.5 μM *Sc*DyPB in 20 mM Tris pH 7.5 (supplemented with 0.1 g L^–1^ BSA and 0.1 M NaCl). The dependency of the rates on the concentration of ABTS shows substrate inhibition by ABTS with the effect being more prominent at low concentration of H_2_O_2_ (Figure 4A, see Figure S2A for the zoom-in of the data at the region of low H_2_O_2_ concentrations). Substrate inhibition by one substrate at a low concentration of the other substrate is a kinetic signature of the enzymes obeying a ping-pong kinetic mechanism such as heme peroxidases. There was no substrate inhibition by H_2_O_2_ at any concentration of ABTS (Figure 4B). However, when incubated with H_2_O_2_ in the absence of ABTS, the *Sc*DyPB was irreversibly inactivated (Figure S3). The rate of inactivation increased with increasing concentration of H_2_O_2_, and the second order rate constants of 4.9 ± 0.3 and 6.2 ± 0.9 M^–1^ s^–1^ were found for H_2_O_2_-driven inactivation of *Sc*DyPB at pH 4.0 and 7.5, respectively (Figure S3).

When the concentration of H_2_O_2_ was treated as a variable, the rate of ABTS oxidation was well in line with the simple Michaelis–Menten equation (Figure S2B and eq S1). However, the dependency of apparent parameters for H_2_O_2_ (*k*_cat_^app^, *K*_M(H_2_O_2_)_^app^, and *k*_cat_^app^/*K*_M(H_2_O_2_)_^app^) on the concentration of ABTS (Figure 4C–E) was more complex than expected for ping-pong peroxidase kinetics with substrate inhibition by ABTS. The dependency of *k*_cat_^app^ (Figure 4C) on [ABTS] does not follow simple saturation with ABTS according to the hyperbola (eq S2) but shows a drop in the parameter value between 0.1 mM and 0.2 mM ABTS. Furthermore, an apparent *k*_cat_/*K*_M(H_2_O_2_)_ does not approach zero with increasing [ABTS] (eq S3) but levels to a constant value of 65.7 ± 2.7 mM^–1^ s^–1^ after the initial drop with increasing [ABTS] (Figure 4E). When the concentration of ABTS was treated as a variable, both the Michaelis–Menten equation and the equation accounting for the substrate inhibition failed to describe the kinetics. The characteristic feature of the kinetics of ABTS oxidation was a slight drop or retardation in the rates observed around ABTS concentrations of 0.1–0.2 mM, which was followed by the increase in rates with a further increase in [ABTS]. This kinetic phenomenon was best revealed in the series made at higher H_2_O_2_ concentrations (Figure 4A).

The simplest kinetic mechanism that can account for the above-described phenomenon assumes the enzyme to be active as two independent, kinetically different forms. One form of the enzyme (form II, E^II^) is a subject of substrate inhibition by ABTS, whereas the other form (form I, E^I^) follows the Michaelis–Menten saturation kinetics. Assuming that the two forms are independent, the rate equation can be written as a sum of the two steady-state rate equations (eq 1)
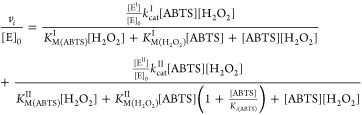
1

In eq 1, the enzyme forms I and II and corresponding kinetic parameters are designated with superscripts I and II, respectively. The enzyme form I follows ping-pong kinetics, whereas form II follows ping-pong kinetics with substrate inhibition by ABTS. This equation was able to account for experimentally observed kinetic peculiarities, like a “kink in the curve” observed after 0.1 mM ABTS (Figure 4A). Global nonlinear regression analysis of the data in Figure 4A according to eq 1 predicted the [E^I^]*k*_cat_^I^/[E]_0_, *K*_M(ABTS)_^I^, and *K*_M(H_2_O_2_)_^I^ values of 59 ± 3 s^–1^, 1.0 ± 0.12 mM, and 0.89 ± 0.06 mM, respectively (for the fit, see Figure S4A). However, because of the interdependency between the parameters, the values of the kinetic parameters for enzyme form II came with a large standard deviation. Precise determination of the parameter values for two different enzyme forms apparently assumes measurements under the experimental conditions where one of the forms is predominant and the contribution by the other is insignificant. Since the activity of *Sc*DyPB was higher (with 1.0 mM ABTS and 0.1 mM H_2_O_2_) when the reactions were started from the working stock made in 50 mM NaAc pH 4.0 (Figure 2C) instead of 20 mM Tris pH 7.5, we also tested the 1.5 μM *Sc*DyPB working stock made in pH 4.0 in making the series with varying [ABTS] and [H_2_O_2_]. Although the general activity was higher, apparent biphasic kinetics persisted also in these experiments, suggesting that *Sc*DyPB existed in two kinetically different forms also in 50 mM NaAc pH 4.0 (data not shown).

In order to evaluate the relative abundancy of enzyme forms, we further assume that the two enzyme forms have the same values of kinetic parameters and they differ only by the presence of substrate inhibition in the case of form E^II^. In this case, eq 1 simplifies to
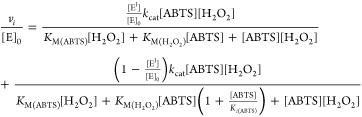
2Global nonlinear regression analysis of the data in Figure 4A according to eq 2 predicted the relative abundancy of forms I ([E^I^]/[E]_0_) and II (1 – [E^I^]/[E]_0_) of 0.18 ± 0.01 and 0.82 ± 0.01, respectively. The estimates of common parameter values for both forms were 327 ± 28 s^–1^, 0.95 ± 0.09 mM, and 0.89 ± 0.06 mM, for *k*_cat_, *K*_M(ABTS)_, and *K*_M(H_2_O_2_)_, respectively. The estimate of the *K*_i(ABTS)_ was 4.12 ± 0.36 μM. Despite having two parameters less, the fitting according to eq 2 was not significantly worse than that according to eq 1 with *R*^2^ values of 0.9957 and 0.9962, respectively (for the comparison of fits, see Figure S4A,B). The high *k*_cat_ value obtained from the analysis according to eq 2 is a result of the low abundancy of the nonsubstrate inhibited form. Despite strong substrate inhibition, the enzyme form E^II^ has significant contribution to the overall activity at [ABTS] below 0.1 mM (Figure S4C,D).

### *Sc*DyPB Exists in Oligomeric Forms with Different Sizes

**Figure 5:**
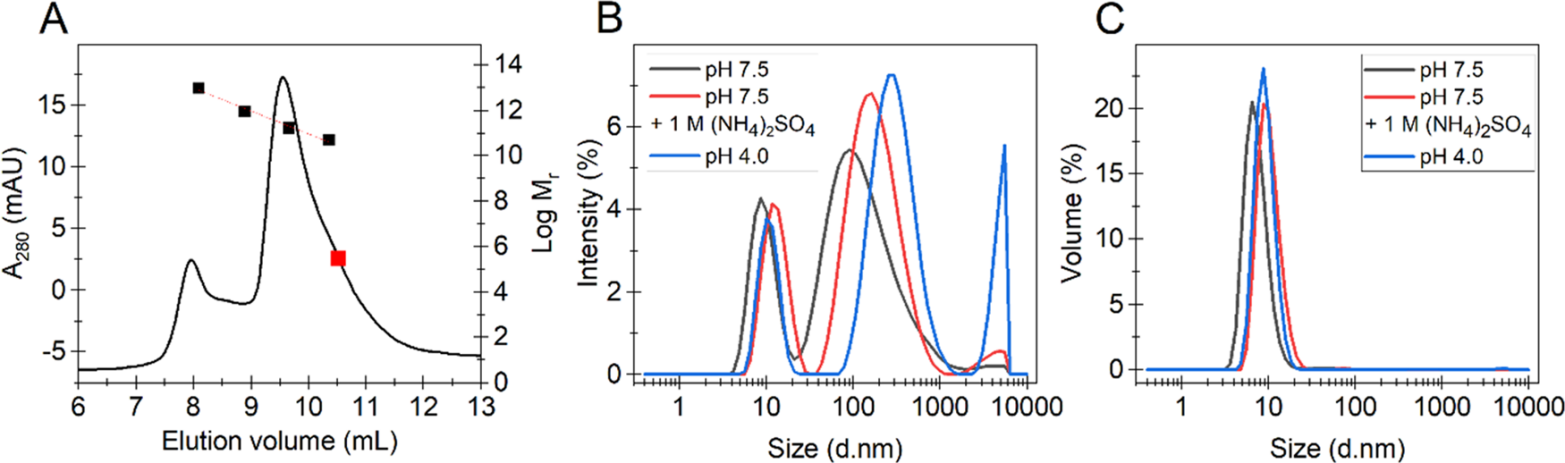
Analysis of the size distribution of *Sc*DyPB. (A) Gel filtration chromatogram of *Sc*DyPB in 20 mM Tris-HCl (pH, 7.5) (supplemented with 100 mM NaCl). Black squares show the elution volume of standard proteins: ferritin (440 kDa), aldolase (158 kDa), conalbumin (75 kDa), and ovalbumin (44 kDa). The red line shows linear regression analysis of the mobility of the standard proteins used for calibration. The red square shows the expected elution volume of the *Sc*DyPB monomer. (B, C) Dynamic light scattering (DLS) analysis of 1.5 μM *Sc*DyPB in 50 mM NaAc pH 4.0, in 20 mM Tris pH 7.5, and in 20 mM Tris pH 7.5 supplemented with 1.0 M ammonium sulfate (as indicated in the plot). Size distribution based on the intensity (B) or volume (C). Traces show the average of at least four consecutive scans.

The results of the kinetic studies described above suggested that *Sc*DyPB may exist as a mixture of different oligomeric forms. Here, we analyzed the size distribution of *Sc*DyPB using gel filtration chromatography and dynamic light scattering (DLS). Analysis using a Superdex-75 column showed that at pH 7.5, the *Sc*DyPB eluted as two peaks. A dominant peak with an apparent molecular weight of 91 kDa and a smaller peak eluted close to the void volume of the column (MW of about 450 kDa) (Figure 5A). Considering the molecular weight of *Sc*DyPB of 34 kDa, the dominant peak corresponds to an apparent trimer. The shoulder in the region of the expected elution of the *Sc*DyPB monomer suggests that the dominant peak may correspond to the mixture of mono- and trimeric *Sc*DyPB. Unfortunately, the gel permeation chromatographic analysis at pH 4.0 but also at pH 7.5 but in the presence of 1.0 M ammonium sulfate was not possible. At pH 4.0, *Sc*DyPB precipitated at high concentrations necessary for this analysis (14.5 μM) and at pH 7.5, but in the presence of 1.0 M ammonium sulfate, the elution of *Sc*DyPB was retarded because of the interaction with the column matrix.

DLS analyses were also applicable at pH 4.0 and 7.5 in the presence of 1.0 M ammonium sulfate. In the case of all conditions tested, the *Sc*DyPB existed as a complex mixture of particles with different sizes with the diameter ranging from 9 nm to more than 4 μm. However, although intensity-based size distribution revealed the presence of large aggregates (Figure 5B), the relative contribution of *Sc*DyPB engaged in these aggregates was less than 1% as judged by the volume-based size distribution analysis (Figure 5C). The majority of *Sc*DyPB appeared in the population of oligomers with an average size of around 10 nm (Figure 5C and Table 1). The lowest average size of *Sc*DyPB oligomers was observed at pH 7.5 followed by pH 4.0 and pH 7.5 but in the presence of 1.0 M ammonium sulfate (Figure 5B,C and Table 1). Although the average size of oligomers depends on which distribution, intensity, or volume was used for the size calculation, the trends were the same. Addition of ammonium sulfate led to the increase in the average size of *Sc*DyPB oligomers, and higher oligomers were observed at pH 4.0 compared to pH 7.5 (Table 1).

**Table 1:** DLS Analysis of the Mean Size and Relative Contribution of *Sc*DyPB Oligomers^a^

experiment conditions	intensity-based size (nm)	volume-based size (nm)	volume-based contribution (%)
pH 7.5	9.8 ± 0.7	7.6 ± 0.3	99.6 ± 0.4
pH 7.5 + 1 M (NH_4_)_2_SO_4_	13.2 ± 0.4	10.4 ± 0.4	99.1 ± 0.2
pH 4.0	10.9 ± 0.4	9.3 ± 0.3	99.3 ± 0.2

a Calculated based on the size distribution of intensity (Figure 5B) and volume (Figure 5C). Mean sizes are calculated from at least four DLS scans, and error bars show SD.

We also recorded the UV–vis spectra of *Sc*DyPB at pH 7.5 and pH 4.0 (Figure S5). At both pH values, there was a clear absorbance of the Soret band around 400 nm, characteristic for heme proteins. Changing the pH from 7.5 to 4.0 resulted in an increase in the absorbance at all wavelengths, but the effect was more prominent at shorter wavelengths, suggesting the contribution of the light scattering. This observation is corroborated by the higher abundancy of large particles observed in DLS spectra at pH 4.0 compared to pH 7.5 (Figure 5B,C). Of note, the pH-dependent changes in the UV–vis spectrum were reversible as the adjustment of pH from 4.0 back to 7.5 restored the initial absorbance spectrum measured at pH 7.5 (Figure S5).

## Discussion

DyP peroxidases often display complex, non-Michaelis–Menten kinetics with substrate inhibition by H_2_O_2_,^21,24,25^ by the reducing substrate,^18,22,23^ or by both.^22^ An apparent positive cooperativity with the reducing substrate has also been observed with many DyP peroxidases.^15,22,27,28^ Kinetics of heme peroxidases are well studied, and they obey a ping-pong kinetic mechanism. Catalysis is initiated by the binding of H_2_O_2_ to the heme in its resting state (Fe^3+^) followed by two-electron oxidation of the heme and formation of the reactive intermediate known as compound I (Cpd I).^58^ Cpd I is reduced back to the resting state via two consecutive one-electron transfer steps from the reducing substrate. Electrons may be transferred directly from the reducing substrate to Cpd I, but many DyPs have shown to employ long-range electron transfer where the reducing substrate is oxidized at surface binding sites.^19,28,59−61^ The latter strategy is used in the oxidation of bulky substrates that cannot pass through the heme access channel(s).

**Figure 6:**
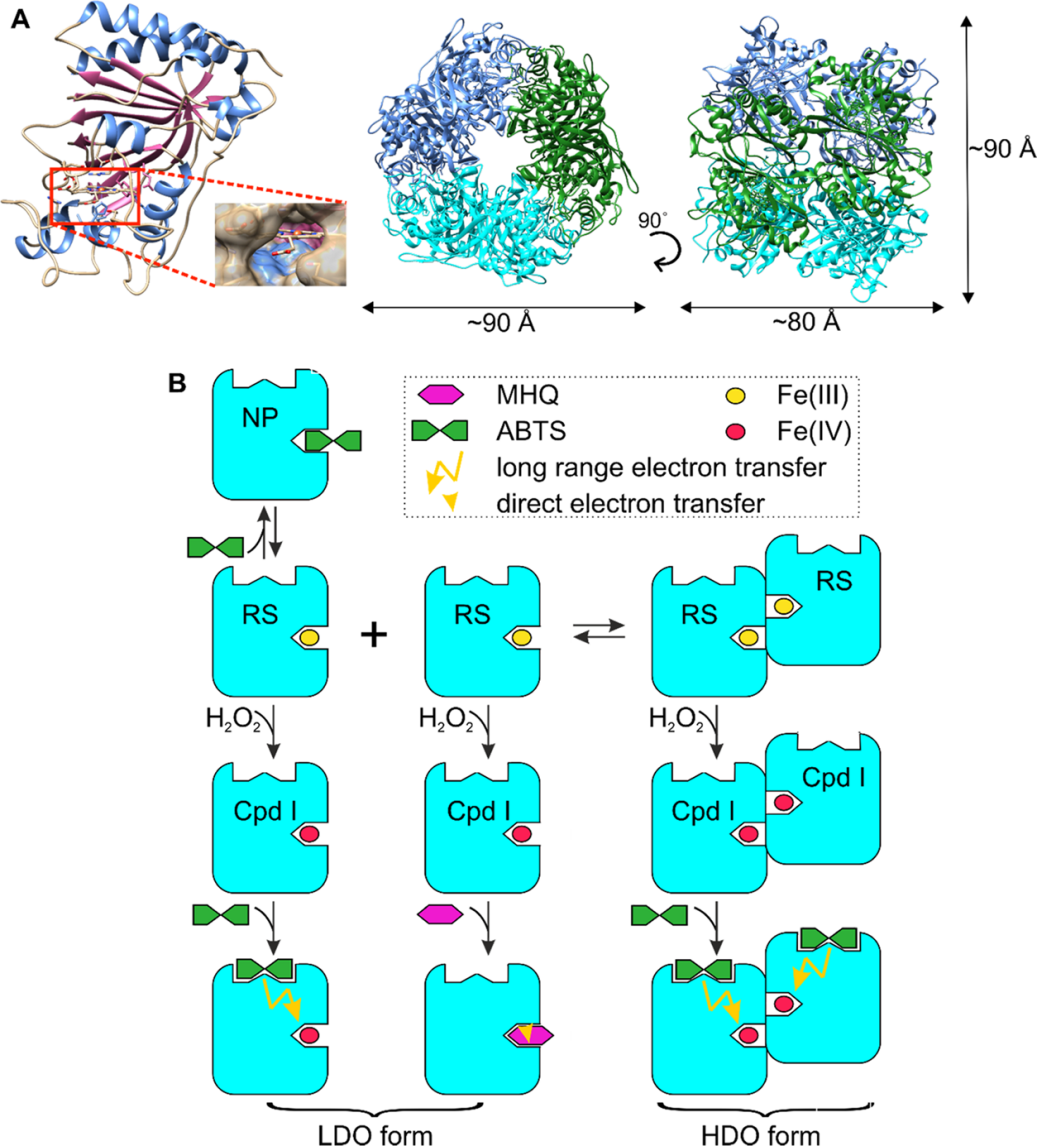
Structure of *Sc*DyPB and simplest possible mechanism of the formation of kinetically different forms of the enzyme. (A) Structure of *Sc*DyPB (PDB: 4GU7). The structure of the *Sc*DyPB monomer, where α- helices, β-sheets, and loops are colored blue, pink, and tan, respectively. The heme is shown as a stick model. Nitrogen, oxygen, and iron atoms are colored blue, red, and brown, respectively. The inset shows the heme access channel of *Sc*DyPB, the propionate pocket (left). The *Sc*DyPB hexamer (trimer of dimers), with dimers shown in different colors and the approximate dimensions of the hexamer (right). (B) Simplest mechanism of the catalysis by *Sc*DyPB that explains the experimental observations of this study. *Sc*DyPB exists as an equilibrium (slow compared with the time frame used for the activity measurements) of the enzyme forms with low (LDO form) and high degrees of oligomerization (HDO form). For the simplicity of visualization of the concept, the LDO- and HDO forms are represented by the monomer and dimer, respectively (in a real system, the degree of oligomerization of both forms is much higher). In their resting state (RS), both forms of the enzyme can be oxidized by H_2_O_2_ to form an active intermediate, compound I (Cpd I). In the case of ABTS as the reducing substrate, the RS is restored by the long-range electron transfer to Cdp I. For MHQ, there is no surface binding site, and Cpd I is reduced via direct electron transfer to the heme iron. The heme access channel is assumed to be inaccessible for the reducing substrate in the enzyme in its HDO form. Therefore, the oxidation of MHQ occurs only with the enzyme in its LDO form. Substrate inhibition by ABTS results from the nonproductive binding of ABTS to the heme (NP) and is possible only with the enzyme in its LDO form. Thus, the conditions that favor the HDO form of *Sc*DyPB will decrease the MHQ oxidizing activity through blockage of the access to the heme and increase ABTS oxidizing through relieving substrate inhibition. Note that possible substrate inhibition by MHQ and oxidation of ABTS through direct electron transfer do not change the general outcome of the model and are omitted for simplicity.

Substrate inhibition is a phenomenon that is often observed with enzymes obeying a ping-pong kinetic mechanism, and it happens when substrates bind to the “wrong form” of the enzyme. In the case of DyP peroxidases, it assumes the binding of the reducing substrate to the enzyme resting state in a way that competes with the binding of H_2_O_2_. The substrate inhibition by H_2_O_2_ occurs when H_2_O_2_ binds to Cpd I and restricts its reduction by the reducing substrate. The *Sc*DyPB studied here was substrate-inhibited by ABTS but not by H_2_O_2_ (Figure 4A,B). Analysis of the structure of *Sc*DyPB (PDB: 4GU7) reveals the presence of one heme access channel, a propionate pocket with a bottleneck radius of 2.45 Å that can possibly accommodate ABTS (Figure 6A). The heme access channel of *Sc*DyPB is similar to the well-studied DtpB of the bacterium *Streptomyces lividans*.^62^ Thus, it seems plausible that substrate inhibition of *Sc*DyPB involves the entrance of the ABTS through the propionate pocket and binding to the heme resting state. Inactivation of *Sc*DyPB upon incubation with H_2_O_2_ (Figure S3) in the absence of ABTS suggests that H_2_O_2_ can interact with Cpd I. Such binding would compete with the direct binding of ABTS to the heme but not with the possible binding to the surface site. In latter case, the oxidation of ABTS would be kinetically favored by the factor of more than 10^4^ (*k*_cat_^app^/*K*_M(H_2_O_2_)_^app^ for the oxidation of ABTS of 65.7 ± 2.7 mM^–1^ s^–1^Figure 4E, versus rate constant for inactivation by H_2_O_2_ of 0.0049 ± 0.0003 mM^–1^ s^–1^, Figure S3) and provides possible explanation for the absence of substrate inhibition by H_2_O_2_.

Characteristic to the enzyme catalyzed reactions, the rate of the oxidation of ABTS and MHQ was proportional to the concentration of the *Sc*DyPB in the reaction (Figure 2A,B). However, we found that the rates of ABTS oxidation were dependent on the concentration of *Sc*DyPB in its working stock used to initiate the reactions. Higher rates were observed at higher concentrations of *Sc*DyPB in the working stock (Figure 2C). The in-depth kinetic characterization of the oxidation of ABTS (Figures 4 and S4) suggests that *Sc*DyPB exists as a mixture of at least two kinetically different enzyme forms, with one being inhibited by ABTS but not the other one. Size exclusion chromatography and DLS analyses revealed that *Sc*DyPB, indeed, exists as a complex mixture of oligomers and aggregates with different sizes (Figure 5). Furthermore, the lower pH (pH 4.0 versus 7.5) and supplementation with ammonium sulfate seemed to favor a higher degree of oligomerization (Figure 5B,C and Table 1). It is also important to note that while *Sc*DyPB working stocks made at pH 4.0 resulted in higher ABTS oxidizing activity when compared to the working stocks made at pH 7.5 (Figure 2A), the opposite was true for the oxidation of MHQ (Figure 2B). Similarly, the presence of 1.0 M ammonium sulfate in the working stock of *Sc*DyPB increased the ABTS oxidizing activity but decreased the MHQ oxidizing activity (Figure 3). Collectively, these results suggest that different mechanisms are used in the oxidation of ABTS and MHQ and that these mechanisms are differently affected by the degree of oligomerization of *Sc*DyPB.

The simplest mechanism that would explain the results of this study is depicted in Figure 6B, and it relies on the four following assumptions. (i) *Sc*DyPB exists as a mixture of two enzyme forms (we note that assuming just two enzyme forms may be an oversimplification, but including more forms leads to the overparametrization of the rate equations that do not permit quantitative analyses) with different degrees of oligomerization. One form has a high degree of oligomerization (HDO form), whereas the other form has a low degree of oligomerization (LDO form). (ii) ABTS can be oxidized at the surface binding site through the long-range electron transfer, but there is no such possibility with smaller substrate MHQ. Oxidation of MHQ takes place in a direct contact with Cpd I (note that there is no need to exclude this possibility for the oxidation of ABTS). (iii) Substrate inhibition by ABTS involves the binding of ABTS to the heme resting state competing with the binding of H_2_O_2_ and Cpd I formation. (iv) Direct access of ABTS and MHQ to the heme and Cpd I is possible only with the LDO form of *Sc*DyPB, but H_2_O_2_ has access to the heme of *Sc*DyPB in its both LDO and HDO forms.

Relative contribution of the HDO form increases with an increasing concentration of *Sc*DyPB in its working stocks. Since the direct access of reducing substrates to heme in the HDO form is blocked, the substrate inhibition by ABTS is relieved, while the oxidation through long-range electron transfer is unaffected. The net result is increased ABTS oxidizing activity with increasing concentration of *Sc*DyPB in its working stocks (Figure 2). Without long-range electron transfer, as proposed for the MHQ substrate, the blockage of the heme access channel in the HDO form will abolish the MHQ oxidizing activity, and it decreases with increasing concentration of *Sc*DyPB in its working stocks (Figure 2). As indicated by the increased average size of *Sc*DyPB oligomers, the lower pH of the *Sc*DyPB working stock as well as the presence of ammonium sulfate in it also seems to increase the relative concentration of the HDO form (Table 1). The result is increased ABTS but decreased MHQ oxidizing activity in the experiments with the *Sc*DyPB working stock made at pH 4.0 (Figure 2) but also at pH 7.5 in the presence of 1.0 M ammonium sulfate (Figure 3).

Long-range electron transfer involves accessible Trp or Tyr residues at the surface of the enzyme. There are two Trp and five Tyr residues in *Sc*DyPB, and all of them, except Tyr 141, are in the solvent-accessible surface. Although the use of surface binding site(s) by *Sc*DyPB remains to be experimentally validated, its ability to oxidize bulky dyes RB4 and RB19 and polymeric lignin^23^ suggests the presence of this possibility. The core dimensions of a *Sc*DyPB (PDB: 4GU7) dimer in the crystal structure are 6 × 8 × 4 nm^3^. Assessment of the structure of *Sc*DyPB using PISA analysis revealed that the most probable multimeric state of *Sc*DyPB would be a hexamer (trimer of dimers) with a buried surface area of 23,290 Å^2^ (27.5% of the total surface area of six monomers, Figure 6A). The hexamer had a diameter of 8 nm × 8 nm × 9 nm (Figure 6A). The maximum particle dimensions *D*_max_ and the radius of gyration *R*_g_ are 86 and 27 Å for the dimer and 99 and 36 Å for the hexamer, respectively, as calculated with GNOM based on the theoretical solution small-angle X-ray scattering curves generated with FoXS.^63,64^ Thus, the *Sc*DyPB population with the diameter of around 10 nm observed in DLS scans (Figure 5B,C and Table 1) may well correspond to a hexamer. However, considering the heterogeneity of enzyme populations revealed in consecutive DLS scans and relatively big differences between intensity- and volume distribution-based sizes (Table 1), identification of this population as a dimer cannot be excluded. Since more than 99% of *Sc*DyPB appears in this peak (Table 1), it is evident that both LDO and HDO forms are merged to this single peak. Unfortunately, our data do not enable us to derive the sizes of LDO and HDO forms separately as we see only an average size of the mixture of different oligomers (Table 1). All in all, the structural as well as biophysical analyses (Figure 5) suggest that *Sc*DyPB exists as a mixture of enzyme populations with different degrees of oligomerization. Our results suggest that lower pH (pH 4.0 compared to pH 7.5) supports the formation of the HDO form. The most probable candidate for changing its protonation state upon shifting from pH 7.5 to 4.0 would be a histidine with a p*K*_a_ value of 6.0–7.0. Although there is one His residue (His 137) in the contact area of adjacent *Sc*DyPB monomers, this residue is not involved in salt bridge formation. Increased contribution of the HDO form in the presence of 1.0 M ammonium sulfate suggests that hydrophobic interactions may be involved in the formation of oligomers. An important assumption of our model was that the heme access channel is not accessible for the reducing substrates, ABTS and MHQ, in the HDO form of *Sc*DyPB (Figure 6B). Similar to the observations made with the oligomerization of other DyPs,^11,35^ the heme access channel in the *Sc*DyPB hexamer is not located in the subunit interface and seems to be accessible. However, a possible blockage of heme access channels upon formation of oligomers with a higher degree of oligomerization that were observed in both gel filtration and DLS analyses (Figure 5) cannot be excluded. Further studies are needed to judge the plausibility of the assumptions underlying the mechanism in Figure 6B.

The biological role of DyP peroxidases and the nature of their native reducing substrates are not known. Many DyP peroxidases are secreted as cargo proteins in the interior of the shell made from encapsulins.^45,46,53,54^ As an example, the DyP of *Mycobacterium smegmatis* is loaded into the encapsulin shell as a dodecamer made from two hexamers.^54^ However, there are no encapsulin-coding genes in the genome of *S. coelicolor*, suggesting that oligomerization of *Sc*DyPB is not related to the packing into encapsulin nanoparticles. Although the exact mechanism and biological role remain to be revealed, the enzyme oligomerization-dependent modulation of the kinetic properties observed here expands the complexity of the kinetics of DyP peroxidases but also suggests novel possibilities for the regulation of their catalytic activity.

## Supporting Information Available

Effects of preincubation time of *Sc*DyPB working stocks before the measurement of the activity with ABTS, kinetics of the oxidation of ABTS, inactivation of *Sc*DyPB by H_2_O_2_, comparison of the fitting of the data of ABTS oxidation according to eqs 1 and 2, contribution of the enzyme forms EI and EII, and absorbance spectra of *Sc*DyPB (PDF)

## Accession Codes

Type B dye-decolorizing peroxidase from *Streptomyces coelicolor*, Q9FBY9.

## Abbreviations

ABTS: 2,2′-azino-di(3-ethyl-benzothiazoline-6-sulfonic acid)
Cpd I: compound I
Cpd II: compound II
DLS: dynamic light scattering
DyP: dye-decolorizing peroxidase
HDO: high degree of oligomerization
LDO: low degree of oligomerization
MHQ: methylhydroquinone
MQ: methylquinone
NaAc: sodium acetate
*Sc*DyPB: type B dye-decolorizing peroxidase of *Streptomyces coelicolor*
