# Supplementary material for: Dye-Decolorizing Peroxidase of Streptomyces coelicolor (ScDyPB) Exists as a Dynamic Mixture of Kinetically Different Oligomers
Source: ACS Omega. 2024 Jan 8;9(3):3866–76. doi: 10.1021/acsomega.3c07963 (PMC10809370; doi:10.1021/acsomega.3c07963)
Supplement: Supplementary file 1 — ao3c07963_si_001.pdf [file ao3c07963_si_001.pdf]

Supporting information to:

Dye-decolorizing peroxidase of *Streptomyces coelicolor* (*Sc*DyPB) exists as a dynamic mixture of kinetically different oligomers

Hegne Pupart[a§], Darja Vastšjonok[b§], Tiit Lukk[a], and Priit Väljamäe[b]*

[a]Department of Chemistry and Biotechnology, Tallinn University of Technology, 15 Akadeemia tee, Tallinn, 12618, Estonia

[b]Institute of Molecular and Cell Biology, University of Tartu, Riia 23b-202, Tartu, 51010, Estonia

*Email: priit.valjamae@ut.ee

[§]H.P. and D.V. contributed equally to this work.

KEYWORDS

dye-decolorizing peroxidase, substrate inhibition, enzyme oligomers, *Streptomyces coelicolor*

Supplementary figures:

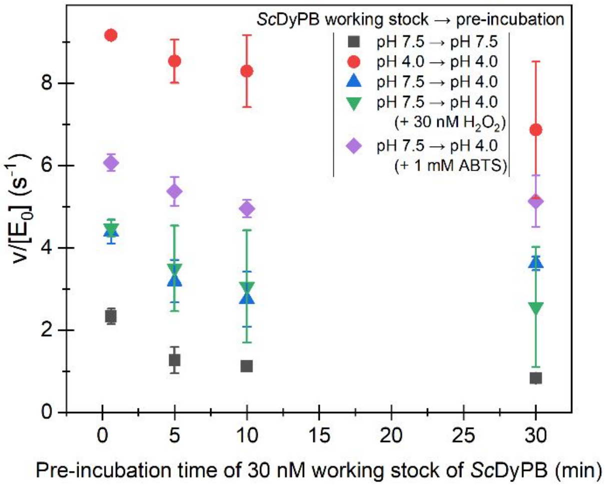

Figure S1. Effects of pre-incubation time of *Sc*DyPB working stocks before the measurement of the activity with ABTS. 1.5 µM *Sc*DyPB working stock (supplemented with 0.1 g L^{-1} BSA) was prepared in NaAc pH 4.0 or Tris-HCl pH 7.5, 0.1 M NaCl buffer. 10 µL of this working stock was added to the cuvette containing 490 µL of 50 mM NaAc pH 4.0 or 5 mM Tris-HCl pH 7.5 (supplemented with 25 mM NaCl) and the resulting 30 nM *Sc*DyPB was pre-incubated at 25 ˚C for selected times before activity measurement with 1 mM ABTS and 0.1 mM H_2O_2. Activity measurements were started by adding 0.5 mL of the mixture of ABTS and H_2O_2 (at appropriate concentrations in 100 mM or 50 mM NaAc pH 4.0 for pre-incubations at pH 7.5 or pH 4.0, respectively) to the cuvette containing 0.5 mL pre-incubated 30 nM *Sc*DyPB. In one series the 30 nM *Sc*DyPB was pre-incubated in the presence of 1 mM ABTS or 30 nM H_2O_2 as shown in the plot. The pH of the 1.5 µM *Sc*DyPB working stock as well as the pH of the pre-incubation mixture is also indicated in the plot. Data are presented as average values (*n* = 3, independent experiments) and error bars show SD.

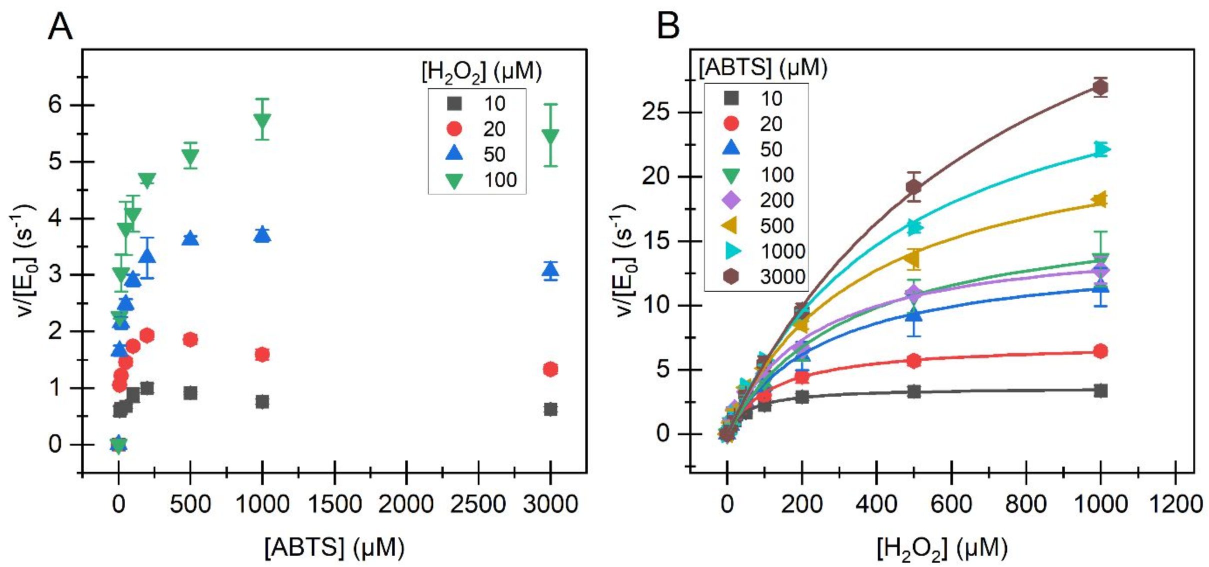

Figure S2. Kinetics of the oxidation of ABTS. Dependency of the initial rates of the oxidation of ABTS on the concentration of (*A*) ABTS and (*B*) H_2O_2. Reactions were made in 50 mM NaAc (pH 4.0) at 25 ˚C. Concentration of *Sc*DyPB was 15 nM and the reactions were initiated by the addition of *Sc*DyPB from the working stock with 1.5 µM *Sc*DyPB in 20 mM Tris pH 7.5 (supplemented with 0.1 g L^{-1} BSA and 0.1 M NaCl). Shown are the same data as in the Figure 3 A and B of the main article but in panel A the data with H_2O_2 concentrations above 100 µM are omitted for better visual inspection of the substrate inhibition by ABTS. Solid lines in panel *B* show non-linear regression of the data according to the Michaelis Menten equation (equation S1 below). The concentration of the substrate that was constant within the series is indicated in the plot. The data are presented as the average values (*n* = 3, independent measurements), and the error bars show the SD.

$$\frac{v_i}{E_0} = \frac{k_{cat}^{app}\,[H_2O_2]}{[H_2O_2] + K_{M(H_2O_2)}^{app}} \quad \text{(S1)}$$

In equation S1 the k_{cat}^{app} and $K_{M}^{app}{}_{(H2O2)}$ stand for the apparent (*i.e* depends on [ABTS]) catalytic constant and Michaelis constant for H_2O_2, respectively. Following relationships between apparent k_{cat} and k_{cat}/K_M for H_2O_2 and the concentration of ABTS are expected for the enzyme obeying ping-pong kinetics with substrate inhibition by ABTS:

$$k_{cat}^{app} = \frac{k_{cat}[ABTS]}{[ABTS] + K_{M(ABTS)}} \quad \text{(S2)}$$

$$\frac{k_{cat}^{app}}{K_{M(H2O2)}^{app}} = \frac{k_{cat}}{K_{M(H2O2)}}\left(\frac{K_{i(ABTS)}}{K_{i(ABTS)} + [ABTS]}\right) \quad \text{(S3)}$$

k_{cat} and $K_{M(H2O2)}$ in equations S2 and S3 are the true catalytic constant and Michaelis constant for H_2O_2, respectively, whereas $K_{i(ABTS)}$ stands for the substrate inhibition constant by ABTS.

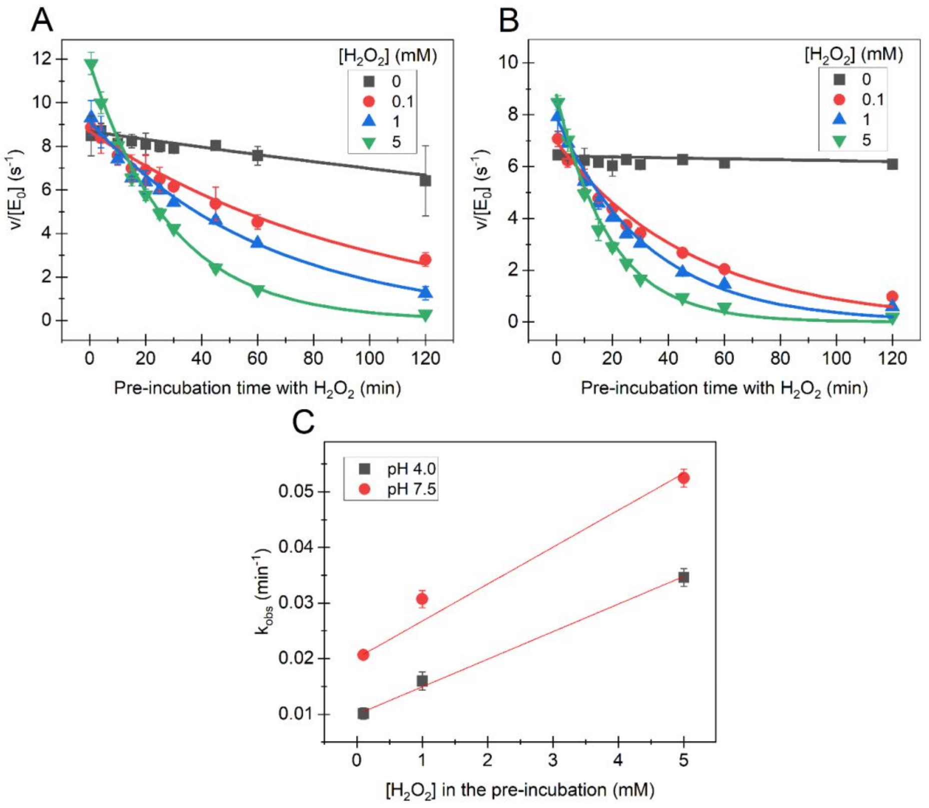

Figure S3. Inactivation of *Sc*DyPB by H_2O_2. 1.5 µM *Sc*DyPB (supplemented with 0.1 g L^{-1} BSA) in (*A*) 50 mM NaAc (pH 4.0) or (*B*) 20 mM Tris-HCl (pH 7.5) was pre-incubated with different concentrations of H_2O_2 (as indicated in the figure) at 25 ˚C. At selected times 10 µL aliquot was withdrawn and added to the cuvette containing 990 µL of the mixture of 1 mM ABTS and 0.1 mM H_2O_2 and the activity was measured by the increase in the absorbance at 420 nm. Solid lines show non-linear regression of the data according to the equation for the first-order reaction (equation S4 below). (*C*) The dependency of the observed first order rate constant of inactivation of *Sc*DyPB (k_{obs}) on the concentration of H_2O_2 in the pre-incubation at pH 4.0 and pH 7.5 (shown in the plot). The dotted line shows linear regression of the data according to the equation S5 below. Data are presented as average values (n = 3, independent experiments) and error bars show SD.

$$\frac{v_i}{E_0} = \left(\frac{v_i}{E_0}\right)_{max} e^{-k_{obs}t} \quad \text{(S4)}$$

In equation S4 the $(v_i/E_0)_{max}$ is the value of v_i/E_0 at pre-incubation time zero and the k_{obs} is the observed first order rate constant for the decay of activity during pre-incubation with H_2O_2. Note that a slight increase in the value of v_i/E_0 in the experiments with high (5 mM) concentration of H_2O_2 in pre-incubation is caused by the increase in the concentration of H_2O_2 in activity measurements.

$$k_{obs} = k_i[H_2O_2] + k_0 \quad \text{(S5)}$$

The k_i and k_0 in equation S5 stand for the second order rate constant of the inactivation by H_2O_2 and first order rate constant of the inactivation in the absence of added H_2O_2, respectively.

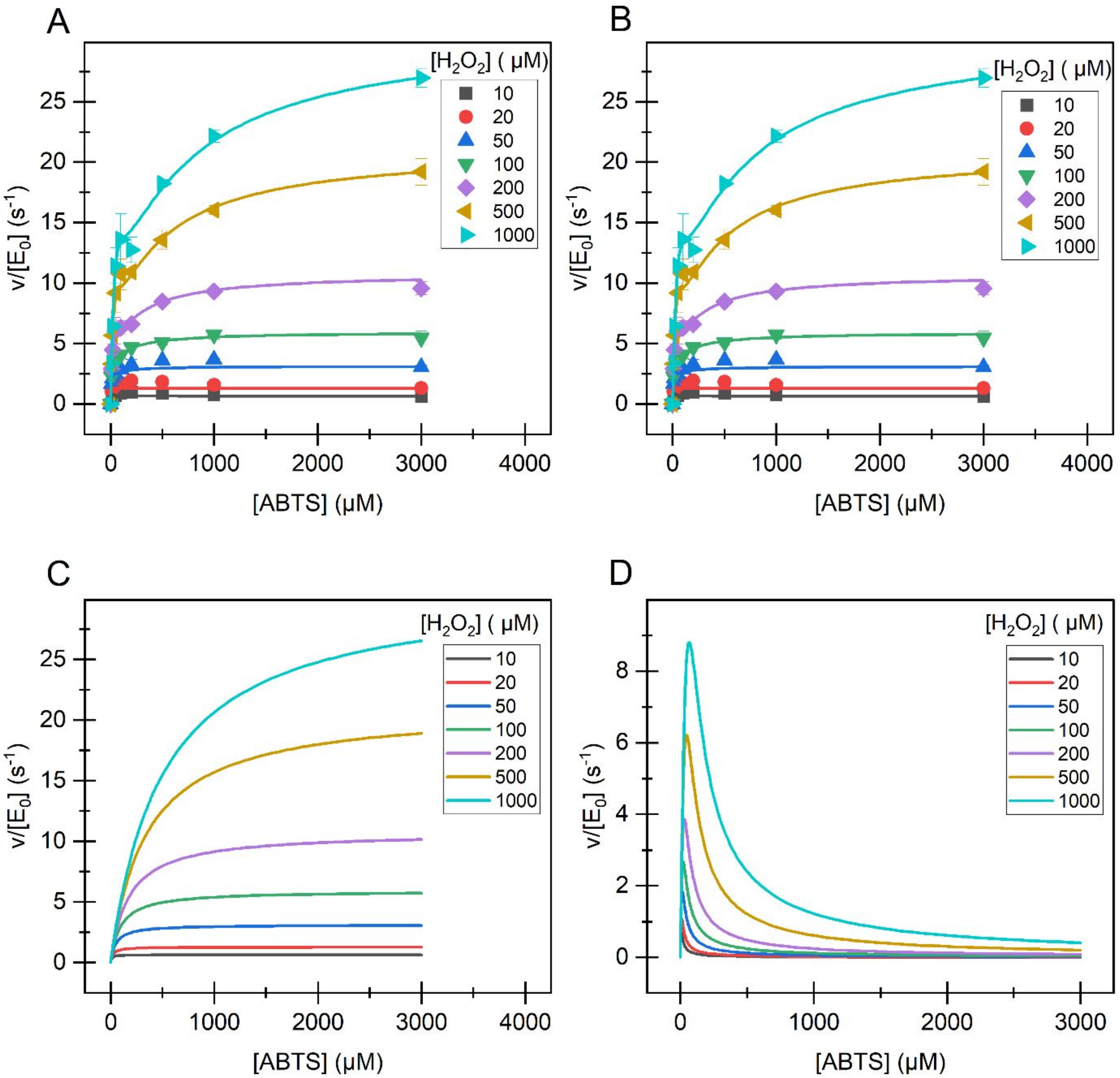

Figure S4. Comparison of the fitting of the data of ABTS oxidation according to the equations 1 and 2, and contribution of the enzyme forms E^I and E^{II}. The experiment data are the same as those in Fig. 4A of the main article. Solid lines show the result of the global non-linear regression analysis according to equation 1 (*A*) or equation 2 (*B*) of the main article. Contribution of the two enzyme forms E^I (*C*) and E^{II} (*D*) in ABTS oxidizing activity. The activity of enzyme forms was calculated using the parameter values obtained from the global non-linear regression analysis according to equation 2 and leftmost and rightmost side of sum in the equation 2 for the form E^I and E^{II}, respectively.

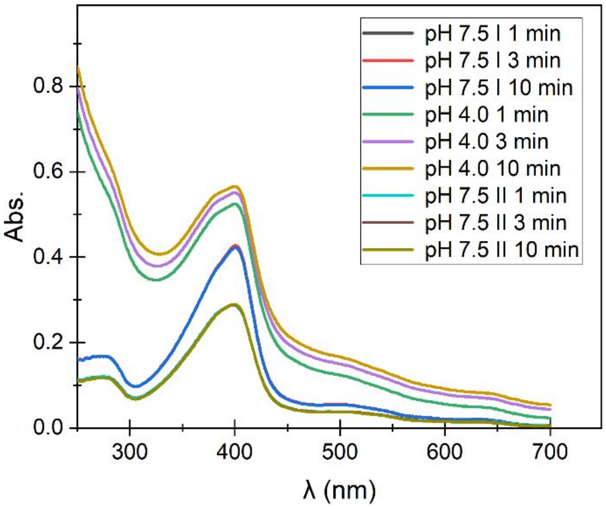

Figure S5. Absorbance spectra of *Sc*DyPB. After recording the spectrum of 3.6 µM *Sc*DyPB in 4 mM Tris-HCl (pH 7.5, supplemented with 20 mM NaCl) in 0.5 mL total volume (designated as pH 7.5 I) the pH was brought to 4.0 by the addition of 26 µL of 1.0 M NaAc (pH 4.0) and spectra were recorded again (designated as pH 4.0). Finally, 150 µL of 1.0 M Tris-HCl (pH 7.5) was added and spectra were recorded (designated as pH 7.5 II). In the case of each condition, the spectra were recorded 1, 3, and 10 min after changing the condition (as indicated in the plot). Note that in the case of pH 7.5 the spectra recorded at different times after changing the condition overlap.
